# Effects of Sport-Specific Training during the Early Stages of Long-Term Athlete Development on Physical Fitness, Body Composition, Cognitive, and Academic Performances

**DOI:** 10.3389/fphys.2017.00810

**Published:** 2017-10-16

**Authors:** Urs Granacher, Ron Borde

**Affiliations:** Division of Training and Movement Sciences, Research Focus Cognition Sciences, University of Potsdam, Potsdam, Germany

**Keywords:** long-term, early sport specialization, motor skills, young athletes, scholastic demands

## Abstract

**Introduction:** Several sports demand an early start into long-term athlete development (LTAD) because peak performances are achieved at a relatively young age (e.g., gymnastics). However, the challenging combination of high training volumes and academic demands may impede youth athletes' cognitive and academic performances. Thus, the aims of this study were to examine the effects of a 1-year sport-specific training and/or physical education on physical fitness, body composition, cognitive and academic performances in youth athletes and their non-athletic peers.

**Methods:** Overall, 45 prepubertal fourth graders from a German elite sport school were enrolled in this study. Participating children were either youth athletes from an elite sports class (*n* = 20, age 9.5 ± 0.5 years) or age-matched peers from a regular class (*n* = 25, age 9.6 ± 0.6 years). Over the 1-year intervention period, the elite sports class conducted physical education and sport-specific training (i.e., gymnastics, swimming, soccer, bicycle motocross [BMX]) during school time while the regular class attended physical education only. Of note, BMX is a specialized form of cycling that is performed on motocross tracks and affords high technical skills. Before and after intervention, tests were performed for the assessment of physical fitness (speed [20-m sprint], agility [star agility run], muscle power [standing long jump], flexibility [stand-and-reach], endurance [6-min-run], balance [single-leg stance]), body composition (e.g., muscle mass), cognitive (d2-test) and academic performance (reading [ELFE 1–6], writing [HSP 4–5], calculating [DEMAT 4]). In addition, grades in German, English, Mathematics, and physical education were documented.

**Results:** At baseline, youth athletes showed better physical fitness performances (*p* < 0.05; *d* = 0.70–2.16), less relative body fat mass, more relative skeletal muscle mass (*p* < 0.01; *d* = 1.62–1.84), and similar cognitive and academic achievements compared to their non-athletic peers. Athletes' training volume amounted to 620 min/week over the 1-year period while their peers performed 155 min/week. After the intervention, significant differences were found in 6 out of 7 physical fitness tests (*p* < 0.05; *d* = 0.75–1.40) and in the physical education grades (*p* < 0.01; *d* = 2.36) in favor of the elite sports class. No significant between-group differences were found after the intervention in measures of body composition (*p* > 0.05; *d* = 0.66–0.67), cognition and academics (*p* > 0.05; *d* = 0.40–0.64). Our findings revealed no significant between-group differences in growth rate (deltas of pre-post-changes in body height and leg length).

**Discussion:** Our results revealed that a school-based 1-year sport-specific training in combination with physical education improved physical fitness but did not negatively affect cognitive and academic performances of youth athletes compared to their non-athletic peers. It is concluded that sport-specific training in combination with physical education promotes youth athletes' physical fitness development during LTAD and does not impede their cognitive and academic development.

## Introduction

The long-term athlete development (LTAD) is a planned, structured and progressive development of youth's athleticism to achieve elite sport success and to engage in lifelong, health-enhancing physical activity (Balyi et al., 2013). Thus, the structured long-term path of athleticism enables talented youth athletes to achieve success on an elite level. In addition, LTAD should be regarded as key for the prevention of chronic diseases (e.g., metabolic syndrome) and as an important instrument to attain physical literacy and to motivate youth for lifetime engagement in sport and physical activity (Lloyd et al., 2015). Expert-based LTAD recommendations foresee a relative late age (i.e., 12–15 years) to start sport-specific training in disciplines like boxing, canoeing, cycling, weightlifting etc. while other sports (e.g., gymnastics, swimming etc.) demand an early start (i.e., 6–9 years) (Bompa, 2000). In those sports with a relatively young peak performance age (e.g., gymnastics, swimming) promising talents initiate sport-specific training often before they finish primary school (American Academy of Pediatrics, 2000; Myer et al., 2015). To achieve successful performance levels in elite sports, high training volumes are required at an early age (American Academy of Pediatrics, 2000). In swimming and gymnastics, training volumes of 6–18 h per week are frequently observed in child athletes (Deutscher Sport Bund, 2006; Feeley et al., 2016). However, early sport specialization has evidence-based side effects like for instance a higher risk of overuse injuries, burnout, and drop out of sports (DiFiori et al., 2014). DiFiori et al. (2014) summarized extrinsic risk factors for overuse injuries and burnout. They identified high training volumes and intensities and frequent competitions as the most important risk factors related to overuse injuries, burnout, and withdrawals from sports in youth athletes (DiFiori et al., 2014). In addition, high training loads in daily training routines at a young age may result in loss of motivation and a lack of concentration (DiFiori et al., 2014). In this context, Richartz and colleagues (Richartz et al., 2005) interviewed 356 German youth athletes (i.e., gymnasts, swimmers, divers, rhythmic gymnasts) with a mean age of 9.7 years. The authors observed that high training loads together with academic demands represent two main factors related to chronic stress in youth athletes (Richartz et al., 2005).

However, for non-athletic youth, there is evidence from a systematic review including cross-sectional and longitudinal studies that higher levels of physical fitness or training-related improvements in physical fitness are associated with better cognitive and academic performances (Donnelly et al., 2016).

To the best of our knowledge, there is no study available that examined the effects of long-term sport-specific training in combination with physical education on physical fitness, body composition, and cognitive as well as academic performances in prepubertal youth athletes compared to their age-matched non-athletic peers. Thus, the aims of this study were to examine the effects of long-term (1 year) sport-specific training and/or physical education on physical fitness, body composition, cognitive and academic performances in a sample of 9–10 year old athletes from sports with relatively young peak performance age compared to age-matched peers. Based on the relevant literature (Richartz et al., 2005; Donnelly et al., 2016), we hypothesized that sport-specific training in combination with physical education enhances physical fitness but may have a negative effect on cognitive and academic development in youth athletes compared to their peers. Of note, knowledge on long-term effects and demands of early sport-specific training and/or physical education on physical fitness and particularly academic performances are crucial for parents and policy makers responsible for the well-being of their children.

## Methods

### Participants

Fourth grade students from a German elite elementary sport school were selected and invited to participate in this study. Local ethical permission was given and all experiments were conducted in accordance with the ethical standards in sports medicine and exercise science (Harriss and Atkinson, 2015). Before the start of the study, parents and teachers were informed about the study purpose and design as well as potential risks. After informed written consent was obtained from all parents or legal representatives, 45 prepubertal children were enrolled in this study. None of the participants suffered from any form of acute musculoskeletal, neurological, or orthopedic disorders that may have affected their ability to execute sport-specific training, physical education, and/or physical fitness tests. Participants were recruited from an elite sports class (ESC) and a regular class (RC, control group) from the same school.

Twenty students from the ESC aged 9.5 ± 0.5 years performed sport-specific training in combination with regular physical education (PE, 3 lessons/week). Four youth athletes of ESC were gymnasts (male/female = 4/0), three trampoline jumpers (male/female = 1/2), three swimmers (male/female = 2/1), four track and field athletes (male/female = 1/3), one BMX cyclist (male/female = 1/0), and five soccer players (male/female = 4/1). In order to cope with athletic and academic demands, a specific training schedule was developed for ESC. Training included three PE lessons per week (Tuesday to Thursday from 7:00 a.m. to 9:30 a.m.) administered in the form of sport-specific training in addition to after-school sport-specific training.

RC included 25 students aged 9.6 ± 0.6 years who performed four regular PE lessons per week that followed the regular PE curriculum of the state of Brandenburg, Germany (i.e., no sport-specific training). Baseline characteristics of the participating students are presented in Table 1.

**Table 1:** Baseline characteristics of the study participants by experimental groups.

	**ESC (*n* = 20)**	**RC (*n* = 25)**
Age (years)	9.5 ± 0.5	9.6 ± 0.6
Sex (m/f)	13/7	13/12
Tanner stages 1/2/3/4/5 (%)		
Pubic hair	95/5/0/0/0	90/10/0/0/0
Genital	53/47/0/0/0	42/42/16/0/0
Years from peak-height-velocity	−3.6 ± 0.2/	−3.1 ± 0.2/
(PHV)	−2.0 ± 0.2	−1.9 ± 0.2
(years; male/female)		

*Values are means and standard deviations. ESC, elite sport class; RC, regular class*.

### Experimental procedure

To assess the effects of sport-specific training and/or physical education on physical fitness, body composition, cognitive and academic performances in youth athletes and their non-athletic peers, a controlled study design with repeated measures (i.e., pre, post) was applied. Pre tests were conducted in January 2014 while our participants attended grade four and post-tests were performed after the 1-year intervention period in January 2015 (grade five). Pre and post-tests of physical fitness and body composition were realized in the local school gym and they were scheduled for the same time of the day (always from 8 am to 11 am). In order not to confound bioimpedance data, subjects were kindly asked to appear in a fasted state on the test day. After the bioimpedance analysis was completed, a rest of 60 min was granted so that subjects were able to have a small breakfast. Physical fitness testing started after the break. Cognitive and academic performance testing was realized at the same daytime on the following day.

### Physical fitness tests

Health and skill-related components of physical fitness were tested using seven single tests from different motor fitness test batteries (Balogun et al., 1997; Stark, 2000; Bös, 2001; Bös et al., 2009; Golle et al., 2015). Speed was assessed by means of the 20-m sprint test, muscle power was evaluated using the 1-kg ball push test (i.e., upper extremities) and the standing long jump test (i.e., lower extremities), agility was tested by means of the star agility run test, flexibility was assessed using the stand-and-reach test, endurance was evaluated with the 6-min run test, and balance was analyzed using the single-leg stance test. Physical fitness tests were instructed by qualified personnel (Master's degree in sports science) using a standardized test protocol. Before testing, all students conducted a 10-min standardized warm-up program consisting of light running followed by different conditioning activities (e.g., side steps, backward running, skipping, submaximal plyometric exercises, and short distance sprints). After the warm-up, each student received standardized verbal instructions and visual demonstration regarding the test procedure. Prior to testing, all students performed one practice trial for each test (except for the 6-min run test) followed by two test trials. The best trial was taken for further analysis.

#### Speed

The 20-m sprint test was applied to assess speed. Participants were instructed to stand in a high starting position with one foot right behind the startling line. Children started on the command “ready-set-go” and accelerated at maximum effort. Time was taken with a stopwatch to the nearest 1/10 s. The 20-m sprint test proved to be reliable in 6–10 year old children with an interclass correlation coefficient (ICC) of 0.73 (Bös et al., 2001).

#### Muscle power

As a proxy of muscle power for the upper extremities, the 1-kg medicine ball push test was applied. Students were instructed to hold a medicine ball in both hands in front of their chest while elbows were on the same level as the hands. From a parallel standing position, students were asked to push the ball as far as possible. The ball pushing distance was documented using a measuring tape to the nearest 10 cm. The ball push test is a reliable test in 8–10 year old children (*r* = 0.82) for the assessment of upper-extremity muscle power (Schulz, 2013).

The standing long jump was used as a proxy of muscle power for the lower extremities. From a parallel standing position and with arms hanging loose to the side, students were instructed to jump as far as possible in horizontal direction and to land on both feet. The jumping distance was documented using a measuring tape to the nearest 1 cm. The standing long jump test proved to be reliable (*r* = 0.96) in 6- to 10-year-olds (Bös et al., 2009).

#### Agility

Agility was tested using the star agility run test (Golle et al., 2015). Students were instructed to run in different running techniques (e.g., forward, backward, side steps) from the center of a 9 x 9-m star-shaped field to the edge and back with four spikes. Time was taken with a stopwatch to the nearest 1/10th of a second. The star agility run test proved to be reliable in 8- to 10-year-olds with an ICC of 0.68 (Schulz, 2013).

#### Flexibility

The stand-and-reach test was applied to analyze flexibility of the lower back and hamstrings (Bös et al., 2009). Students performed the test barefooted and with extended legs and feet close together while standing on an elevated platform. Subjects were asked to bend over using their maximal range-of-motion. Knees, arms, and fingers were fully extended for at least 2 s during the test. A tape measure was attached to the platform with 100 cm corresponding to the upper level of the platform. If students were able to reach beyond their toes, values above 100 cm were measured (i.e., good flexibility). Values below 100 cm indicated that the person was not able to reach the toes (i.e., limited flexibility). In 7–11 years old children, the stand-and-reach test is a reliable test (*r* = 0.86) for the assessment of flexibility (Bös et al., 2009).

#### Endurance

Endurance was assessed using the 6-min run test (Bös et al., 2009). Students were instructed to run as far as they could on a 54 m circuit in the gym over a time period of 6 min. Split times were provided after 3 and 5 min. The maximal distance achieved during the 6-min run test was used for further data analysis. With an ICC of 0.86 the test proved to be reliable in children aged 6–10 years (Bös, 2001; Bös et al., 2009).

#### Balance

Balance was tested using the single-leg stance test (Balogun et al., 1997). The dominant leg of our participants was determined with the help of the modified Lateral Preference Inventory Questionnaire (Coren, 1993). Students were asked to stand barefooted in a single-leg stance position with eyes opened. The non-dominant foot was placed against the inside of the dominant leg (i.e., knee) and hands were held akimbo. Students were instructed to stand as long as they could and as quiet as possible during the test, but not longer than 180 s. The test was terminated if students moved their arms or feet in order to achieve stability or if test-operator intervention was required. Time was taken with a stopwatch to the nearest 1/10th of a second. The single-leg stance test is a reliable test (*r* = 0.95) to analyze balance (Balogun et al., 1992).

### Anthropometrics and body composition

Sitting and standing body height were tested without shoes to the nearest 0.5 cm using a stadiometer (seca 217, Seca, Hamburg, Germany). Biological maturity was estimated by means of assessing years from peak-height-velocity (PHV) derived from sitting and standing body height, body mass, and age (Mirwald et al., 2002). In general, children and adolescents can be classified into three categories according to their maturity status: pre-PHV (−3 years to > −1 years from PHV), circa/around PHV (−1 to +1 years from PHV), and post-PHV (>1 to +3 years from PHV) (Mirwald et al., 2002). Sexual maturation was determined using Tanner stages (Marshall and Tanner, 1969, 1970). In this regard, parents or legal representatives were asked to choose from photographs and descriptions of the five Tanner stages the stage that was most similar to the present maturation status of their child. Tanner stages were recorded for genital and pubic hair for boys and breast and pubic hair for girls (Marshall and Tanner, 1969, 1970).

A non-invasive bioelectrical impedance analysis system (BIA, InBody 720, BioSpace, Seoul, Korea) was applied to assess students' body composition. According to published BIA guidelines (Shafer et al., 2009), students were instructed to abstain from exercise 12 h prior to testing. Outcome variables included body mass (kg), body mass index (kg/m^2^), relative skeletal muscle mass (%), and relative body fat mass (%). Of note, Tompuri et al. (2015) observed excellent agreements between DEXA and BIA (i.e., InBody 720) for lean body mass in children aged 7.7 ± 0.4 years (ICC: girls = 0.93, boys = 0.92).

### Cognitive and academic performance tests

To evaluate cognitive and academic performances, four tests were applied which included the ELFE 1–6 reading test, the DEMAT 4 mathematics test, and the HSP 4–5 spelling test for the assessment of academic performances as well as the d2-test to evaluate cognitive function (i.e., attentional capacity and concentration). The test developer provided age-specific reference values for all tests. In addition, grades in German, Mathematics, English, and PE were documented and used for further analysis (1 = very good, 6 = unsatisfactory).

#### Reading

The ELFE 1–6 test was used to evaluate reading performance (Lenhard and Schneider, 2006). The test comprises three parts: (i) word understanding (max. 72 points), (ii) sentence understanding (max. 28 points), and (iii) text understanding (max. 20 points).

The sum score of all three parts (max. 120 points) was used for further data analysis. The ELFE 1–6 proved to be reliable with an ICC of 0.91 in primary school children. (Galuschka et al., 2015).

#### Mathematics

To examine performance in mathematics, the DEMAT 4 test was applied for fourth graders (Gölitz et al., 2006). This test consists of three test items: i) arithmetic tasks (max. 19 points), written math tasks (max. 14 points), and geometric tasks (max. 7 points). The sum score of all three items (max. 40 points) was used for further data analysis. High reliability was reported for the DEMAT 4 test in 4th graders with an ICC of 0.87 (Gölitz et al., 2006).

#### Spelling

Our students spelling ability was tested using the HSP 4–5 test (May, 2012). For this purpose, students were instructed to write dictated words and sentences on a test sheet. The final score (max. 42 points) was taken from the number of words that were spelled correctly. The HSP 4–5 test showed excellent reliability with an ICC of 0.92 in children aged 9–10 (May, 2012; Galuschka et al., 2015).

#### Attention and concentration

The d2-test was applied to evaluate selective attention and concentration of our participants (Brickenkamp et al., 1998). The total score (max. 658 characters) was calculated by subtracting the errors from the total number of edited characters (Brickenkamp et al., 1998). The d2-test is a reliable test (*r* = 0.95) to analyze selective attention and concentration in 9–60 year olds (Brickenkamp et al., 1998).

### Documentation of sport-specific training and/or physical education

During the intervention period, relevant training modalities such as training volume and intensity were documented for every single sport-specific training session and PE lesson by the responsible teacher and coach. The documented information comprised the type of training (i.e., PE or sport-specific training), training volume (i.e., frequency [PE classes and/or sport-specific training sessions per week], duration of PE classes and/or sport-specific training sessions), main training content of each session (e.g., strength-oriented, endurance-oriented, speed-oriented, technical skills), and number of participating students (i.e., adherence) (Castelli et al., 2014). For the assessment of training intensity, a perceived exertion scale was used for children. The scale contained verbal expressions along a numerical response range of 0 (i.e., very very easy) to 10 (i.e., very very hard) (Faigenbaum et al., 2004). A recently published meta-analysis indicated that the use of perceived exertion scales for children is a valid means to record training intensity with weighted correlation coefficients ranging from 0.84–0.87 between perceived exertion scales for children and physiological outcome measures (e.g., heart rate, oxygen uptake) (Rodriguez et al., 2016). During each lesson or training session, teachers were instructed to ask five randomly selected students for their perceived exertion. The mean score of the five students was used for further analysis.

### Statistical analyses

Normal distribution of data was examined using the Shapiro-Wilk test. If normal distribution was confirmed, descriptive data were presented as group means and standard deviations (SD) otherwise as median and interquartile range. Between-group baseline differences were assessed using either independent sample *t*-tests for interval scaled data or non-parametric Mann-Whitney-U tests for ordinal scaled data. To examine differences between classes at post-test, either an analysis of covariance (ANCOVA) for interval scaled data or the non-parametric Quade test was applied with baseline data entered as covariate. In addition, in a within- and between-subject approach, pre-post-changes in body height and leg length were computed for each participant. Using an independent sample *t*-test, between-group differences from deltas in body height and leg length were calculated to examine whether growth speed/rate differently affected the two experimental groups over the course of the intervention period. Our findings revealed no significant.

The level of significance was set at *p* < 0.05. Effect sizes were calculated by converting partial eta-squared to Cohen's *d* (Cohen, 1988). The effect size is a measure of the effectiveness of a treatment and it helps to determine whether a statistically significant difference is a difference of practical concern. According to Cohen (1988), effect sizes can be classified as small (0.00 ≤ *d* ≤ 0.49), medium (0.50 ≤ *d* ≤ 0.79), and large (*d* ≥ 0.80) (Cohen, 1988). The SPSS 23.0 package (SPSS Inc., Chicago, IL, USA) was used for statistical analyses.

## Results

All participating students received their intervention as allocated. Only one student from the ESC dropped out after baseline testing because his parents moved to a different city. Overall, 45 students completed the study (*n* = 20 ESC, *n* = 25 RC). None of the participating students reported any test- or training-related injuries over the 12 months intervention period. No significant baseline differences were found between classes in terms of age, sex, Tanner stages, and time to PHV (Table 1).

### Anthropometrics, body composition, and physical fitness

In terms of anthropometrics, all students were classified as pre-PHV (Table 1). At baseline, significant between-group differences were found for body height, body mass, BMI, and body composition (all *p* < 0.05, *d* = 1.13–1.84). More specifically, participants in the ESC were shorter (3%), lighter (31%), had a lower BMI (22%), more relative skeletal muscle mass (6%), and less relative body fat mass (14%) (Table 2). After the intervention period, significant between-group differences were found for body height, body mass, and BMI. However, no significant between-group differences were examined for relative body fat mass and relative skeletal muscle mass (Table 3).

**Table 2:** Baseline values of anthropometrics, body composition, physical fitness, cognitive and academic performance.

	**Variables**	**ESC**		**RC**		**Δ %**	***P-*value (*d*)**
		***M***	***SD***	***M***	***SD***		
Anthropometrics, body composition	Body height [cm]	140.5	5.7	144.7	6.2	3	<0.05 (0.68)
	Body weight [kg]	32.0	6.0	41.9	10.3	31	<0.05 (1.11)
	Body Mass Index [kg/m^2^]	16.2	2.4	19.7	3.4	22	<0.01 (1.13)
	Relative skeletal muscle mass [%]	46.5	3.0	40.4	4.1	6	<0.01 (1.62)
	Relative body fat mass [%]	10.2	6.2	24.0	8.0	14	<0.01 (1.84)
Physical fitness	20-m sprint [s]	3.9	0.2	4.3	0.4	10	<0.01 (1.16)
	1-kg ball push [m]	4.7	0.7	4.4	0.7	6	n.s.
	Standing long jump [m]	1.5	0.1	1.3	0.3	19	<0.01 (1.20)
	Star agility run [s]	19.5	1.3	22.3	1.7	14	<0.01 (1.77)
	Stand-and-reach [cm]	108.8	9.3	103.6	4.8	5	<0.05 (0.70)
	6-min run [m]	1233.5	97.5	1012.4	100.2	18	<0.00 (2.16)
	Single-leg stance [s]	104.6	55.6	83.9	57.8	20	n.s.
Cognitive/academic performance	ELFE 1–6 (max. 120 points)^*^	71.0	62–81	76.0	50–93	7	n.s.
	DEMAT 4 (max. 40 points)^*^	15.0	12–20	17.0	11–21	12	n.s.
	HSP 4–5 (max. 42 points)^*^	24.0	16–32	30.0	23–36	20	n.s.
	d2-test^*^	290.5	263–333	313.0	277–372	7	n.s.
	German grade	2.0	2–2	2.0	2–3		n.s.
	Mathematics grade	2.0	2–3	2.0	2–3		n.s.
	English grade	2.0	1–3	2.0	1–3		n.s.
	PE grade	1.0	1–2	3.0	2–3		<0.01 (1.51)

* *Values are medians and interquartile ranges. d, Cohen's d effect size; ESC, elite sport class; M, mean; n.s., non-significant; PE, physical education; RC, regular class; SD, standard deviation*.

**Table 3:** Adjusted post-test values of anthropometrics, body composition physical fitness, cognitive and academic performances.

	**Variables**	**ESC**		**RC**		**Δ %**	***P-*value (*d*)**
		***M***	***SD***	***M***	***SD***		
Anthropometrics, body composition	Body height [cm]	148.3	6.3	149.6	7.5	1	<0.01 (1.23)
	Body mass [kg]	37.1	7.9	48.1	15.6	23	<0.05 (0.80)
	Body Mass Index [kg/m^2^]	17.3	2.9	20.7	4.8	16	<0.05 (0.79)
	Relative skeletal muscle mass [%]	45.5	3.5	41.7	5.0	4	n.s.
	Relative body fat mass [%]	14.6	7.1	22.1	10.0	8	n.s.
Physical fitness	20-m sprint [s]	4.1	0.2	4.5	0.4	9	<0.05 (0.77)
	1-kg ball push [m]	5.1	0.8	5.1	0.8	0	n.s.
	Standing long jump [m]	1.6	0.1	1.4	0.2	13	<0.05 (0.90)
	Star agility run [s]	19.0	1.4	20.6	1.9	8	<0.05 (0.77)
	Stand-and-reach [cm]	105.4	8.8	97.0	7.8	9	<0.01 (1.40)
	6-min run [m]	1214.9	153.2	1076.0	97.7	13	<0.05 (0.75)
	Single-leg stand [s]	139.2	59.1	68.6	60.4	103	<0.01 (1.22)
Cognitive/academic performance	ELFE 1–6 (max. 120 points)^*^	102.5	86–112	108.0	86–110	5	n.s.
	DEMAT 4 (max. 40 points)^*^	22.5	16–29	17.5	15–23	22	n.s.
	HSP 4–5 (max. 42 points)^*^	33.0	29–36	32.5	27–35	2	n.s.
	d2-test^*^	498.0	393–552	418.0	401–481	16	n.s.
	German grade^*^	2.0	2–2	2.0	2–3		n.s.
	Mathematics grade^*^	2.0	1–3	3.0	2–3		n.s.
	English grade^*^	2.0	2–3	2.0	2–3		n.s.
	PE grade^*^	1.0	1–1	3.0	2–3		<0.01 (2.36)

* *Values are medians and interquartile ranges d, Cohen's d effect size; ESC, elite sport class; M, mean; n.s., non-significant; PE, physical education; RC, regular class; SD, standard deviation*.

Table 2 illustrates group specific physical fitness, anthropometrics, and body composition data at baseline. Our analyses indicated significantly better performances at baseline in the ESC compared to the RC in five (i.e., 20-m sprint test, standing long jump test, star agility run test, stand-and-reach test, 6-min run test) out of seven physical fitness tests (Δ5–19%, *p* < 0.05, *d* = 0.70–2.16). No between-group baseline differences were found for the 1-kg ball push and the single-leg stance test. After the intervention period significant between-group differences were observed in six out of seven test items (Δ8–103%, *p* < 0.05, *d* = 0.75–1.40) in favor of the ESC (Table 3). However, the ANCOVA analysis did not detect a between-group difference at post for the 1-kg ball push test (Δ0%, *p* > 0.05). No statistically significant between-group differences were detected for deltas in body height (*p* = 0.945) and leg length (*p* = 0.498).

### Cognitive and academic performance

At baseline, no significant between-group differences were found for all analyzed cognitive and academic parameters (Table 2). After completion of the intervention period, the non-parametric Quade test revealed no significant between-group differences in measures of cognitive and academic performance (*p* > 0.05). In addition, post-intervention, no significant between-group differences were found for grades in German, Mathematics, and English (*p* > 0.05). The PE grade was significantly better in the ESC compared to the RC at post-test (*p* < 0.01, *d* = 2.36). Even though our analyses revealed no significant between-group differences post-intervention in measures of cognitive and academic performances, ESC showed higher scores in all cognitive and academic measures (Δ5–22%, *p* > 0.05, *d* = 0.40–0.64) (Table 3).

### Documentation of training data

**Figure 1:**
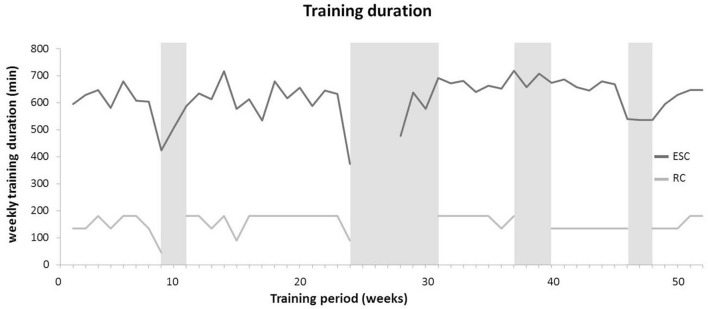
Weekly duration of sport-specific training and physical education for both classes. Gray bars symbolize periods of school holiday. While no physical education classes were conducted in the ESC during school holidays, sport-specific training was performed. *ESC*, elite sport class; *RC*, regular class.

**Figure 2:**
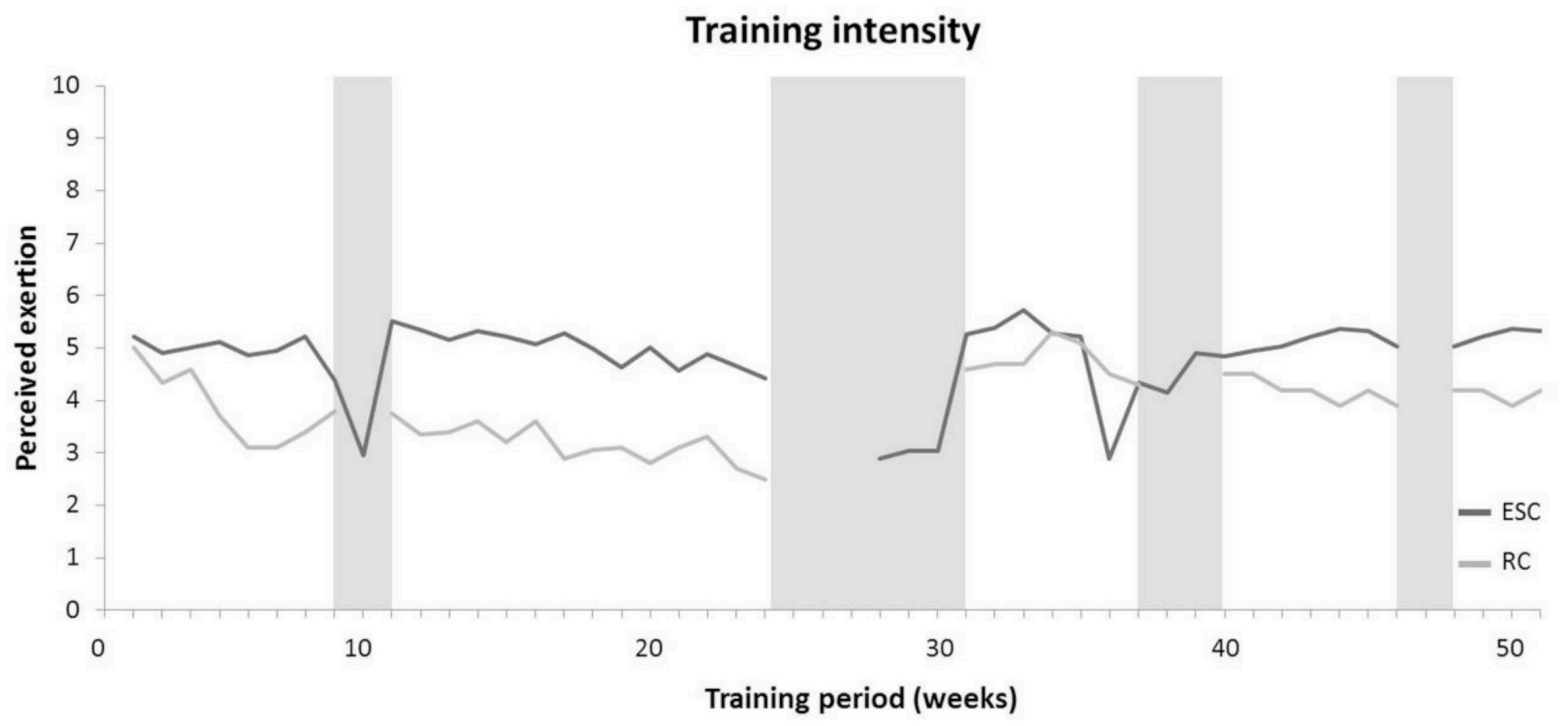
Weekly intensity of sport-specific training and physical education for both classes. Gray bars symbolize periods of school holidays. While no physical education classes were conducted in the ESC during school holidays, sport-specific training was performed. *ESC*, elite sport class; *RC*, regular class.

Training and PE data for the ESC and the RC are presented in Table 4. Subjects in the ESC realized significantly more sport-specific training sessions and PE classes per week (8.2 vs. 3.4 sessions/week, *p* > 0.05, *d* = 2.56) compared to the RC over the 1-year intervention period. Further, the total duration of sport-specific training sessions and PE lessons per week was significantly larger in ESC compared to RC (620 vs. 155 min/week, *p* > 0.05, *d* = 2.50) over the 1-year training period. The average weekly duration of ESC sport-specific training and physical education is illustrated in Figure 1. In terms of ESC training contents, coaches and PE teachers reported technical skill training as the main training content in both, sport-specific training and PE lessons with an average of 390 min/week. Thus, the relative part of movement skill training in ESC amounted to 63% of the entire training volume. In the RC group, endurance training comprised 33% of the overall PE duration and was therefore top ranked in terms of training contents followed by movement skill training (27%). Figure 2 indicates a significantly higher training intensity in the ESC compared to the RC with 4.8 vs. 3.4 points on the visual analog scale ranging from 0 to 10 (*p* < 0.05, *d* = 1.39). Students of both classes showed high adherence rates in PE lessons and sport-specific training with no significant between-group differences (95.9 vs. 95.6%, *p* > 0.05, *d* = 0.28).

**Table 4:** Documentation of sport-specific training and physical education for both classes.

**Variables**	**ESC**			**RC**	**Δ %**	***P-*value (*d*)**
	**PE**	**SST**	**total**	**PE**		
**TRAINING VOLUME**						
Training frequency [ø TS/week]	2.7	5.5	8.2	3.4	141	<0.01 (2.56)
Training duration [ø min/week]	120	500	620 (100%)	155 (100%)	333	<0.01 (2.50)
**TYPE OF TRAINING [ø min/week]**						
Strength	25	50	75 (12%)	30 (20%)	150	n.s.
Endurance	20	40	60 (10%)	55 (33%)	10	n.s.
Speed	40	30	70 (11%)	30 (20%)	133	n.s.
Coordination	5	20	25 (4%)	0 (0%)		
Technique	30	360	390 (63%)	40 (27%)	975	n.s.
**TRAINING INTENSITY**						
Perceived exertion [0 = very, very easy; 10 = very, very hard]	3.1	5.1	4.8	3.4	41	<0.01 (1.39)
**COMPLIANCE**						
Adherence [%]	97.0	95.2	95.9	95.6	0	n.s.

*d, Cohen's d effect size; ESC, elite sport class; n.s., non-significant; PE, physical education; RC, regular class; SST, sport-specific training; TS, training sessions*.

## Discussion

This is the first study to evaluate the effects of long-term sport-specific training and/or physical education on physical fitness, body composition, cognitive and academic performances in youth athletes and their age-matched peers. The main findings of this study were: (i) at baseline, ESC compared to RC showed better performances in physical fitness, less relative body fat mass and more relative skeletal muscle mass but no significant differences in cognitive or academic performances; (ii) mean training volume and intensity over the 1-year intervention period were significantly higher in the ESC compared to the RC; (iii) high sport-specific training and physical education volumes proved to be feasible and safe (no training-related injuries) with high adherence rates (96%) in the ESC; (iv) better performance levels in physical fitness were maintained or even enlarged in the ESC compared to the RC over the course of the 12 months intervention period; (v) high volume sport-specific training and physical education did not have any negative effects on cognitive or academic development in the ESC compared to the RC. Given that we controlled for potential between-group differences at baseline and that we did not detect any significant between-group difference in deltas of body height and leg length, it appears legitimate to ascribe the observed effects to sport-specific training and/or physical education and not to growth speed/rate.

### Physical fitness, anthropometrics, and body composition

At baseline, ESC showed better performances in physical fitness, higher relative skeletal muscle mass and less relative body fat mass compared to RC. These findings could be the result of an effective talent identification program that enabled the selection of children with better performances compared to their age-matched peers. In fact, the state of Brandenburg, Germany initiated a statewide talent identification program in 2009 (www.emotikon-grundschulsport.de). Each year, between 14,000 and 16,000 children (3rd graders) are tested for their physical fitness in Brandenburg's primary schools (Golle et al., 2015). Children in the ESC were selected based on the results of the talent identification program and through specific recommendations from coaches and PE teachers. Another explanation of between-group baseline differences in physical fitness and body composition might be ESC's participation in sport-specific training right at the beginning of the 4th grade. In other words, ESC students began their sport-specific training 6 months prior to the commencement of this study. Maturational factors did not influence the observed between-group differences in physical fitness and body composition because we did not find any baseline differences in biological maturity between ESC and RC (Goncalves et al., 2012). In summary, it seems that the early start into LTAD and the talent selection program to attend the sport-specific class but not maturation were the main reasons to explain baseline superiority in physical fitness and body composition in the ESC compared to the RC.

After the 12 months intervention period, ESC students showed larger improvements in physical fitness (i.e., 20-m sprint test, standing long jump test, star agility run test, stand-and-reach test, 6-min run test, single-leg stance) after having participated in regular PE and additional sport-specific training compared to RC. These findings are supported by recently published articles (Vanttinen et al., 2011; Drenowatz et al., 2013; Golle et al., 2014; Granacher et al., 2016). For example, Vanttinen et al. (2011) examined changes in physical fitness and body composition over the course of two seasons in 11 year old soccer players compared to an age-matched control group. The results showed that parameters of physical fitness and body composition of youth soccer players were better than those of the controls, especially in speed (i.e., 10 and 30-m sprint), agility (i.e., Figure 8 run), lower leg muscle power (i.e., countermovement jump), endurance (i.e., VO_2_max, shuttle run test), and percentage of body fat. The authors concluded that soccer training resulted in additional behavioral and physiological adaptations which are beyond growth and maturation. Further, Golle et al. (2014) reported significantly better performances in physical fitness tests for children (aged 9–12 years) who continuously participated in sport clubs compared to age-matched peers who did not attend sports club. In another study, it was shown that grade two students participating in organized sport for more than once a week achieved better physical fitness levels and were less likely to be overweight compared to their non-participating peers (Drenowatz et al., 2013).

### Cognitive and academic performances

Besides the positive effects of sport-specific training in combination with physical education on physical fitness in the ESC, no significant between-group differences were detected for measures of cognitive and academic performances at baseline and post-tests. The lack of training effects on cognitive and academic performances in the ESC compared to the RC is not in agreement with the existing literature. For instance, a systematic review and meta-analysis (Fedewa and Ahn, 2011) examined the effects of physical activity and physical fitness on cognitive and academic performances in 5–16 year old children. Fifty nine cross-sectional and longitudinal studies were included in the analysis (Fedewa and Ahn, 2011). The aggregated findings indicated small but significant effects of physical activity and physical fitness on childrens' cognitive and academic performances with an effect size of 0.32 (Hedge's *g*, 95% confidence interval [CI]: 0.26–0.37, *p* < 0.01) for cross-sectional studies and an effect size of 0.35 (Hedge's *g*, 95% confidence interval [CI]: 0.26–0.43, *p* < 0.01) for longitudinal studies (Fedewa and Ahn, 2011). In accordance with these findings, other systematic reviews of cross-sectional studies showed large positive associations between physical fitness and cognitive as well as academic performances in school-aged children (Chaddock-Heyman et al., 2014; Donnelly et al., 2016). One possible explanation for the lack of training effects on cognitive/academic performances in the present study is the challenging combination of high training volume together with academic demands which may have resulted in stress and academic overload (Richartz et al., 2005). In fact, it has previously been reported that sport-specific demands (e.g., high training volumes, large number of competitions) could be responsible for chronic stress and even burnout (Malina, 2010). These psychological problems together with symptoms such as fatigue, depression, loss of motivation, and lack of concentration may result in a decline in cognitive and academic performances (Kusurkar et al., 2013; Wagner et al., 2015; Rabiner et al., 2016). Interestingly, in the present study, no significant between-group differences were found in terms of cognitive and academic performances at pre and post-test. Nevertheless, it can be postulated that ESC's cognitive and academic performances were not affected by the high training volume. In other words, the observed high training volume of 8.2 training sessions per week with a total duration of 620 min per week at moderate training intensities had no negative effects on the achievements in standardized cognitive and academic tests as well as on school grades in the ESC compared to the RC.

### Documentation of training data

The training documentation revealed higher training volumes and intensities in the ESC compared to the RC. The contents of the sport-specific training were part of the structured and planned pathway during LTAD. Of note, LTAD consists of seven sequential stages (1. Active Start, 2. FUNdamentals, 3. Learn to Train, 4. Train to Train, 5. Train to Compete, 6. Train to Win, 7. Active for Life) and considers individual maturation level rather than chronological age (Balyi et al., 2013; Granacher et al., 2016). In the present study, ESC students can be categorized in the stage “Learn to Train” that is characterized by a pre-PHV maturation status (>-1 years from PHV) (Lloyd et al., 2015). Sport-specific training of this study was conducted with reference to the “Learn to train” stage of the LTAD. Therefore, coaches implemented movement skill training as the main part of sport-specific training and physical education (63% of the entire training time). Moreover, a recent study examined the time per week spent in organized sports of 1,190 youth athletes aged 7–18 years. The reported time of 636 min per week spent in organized sports was comparable with the findings of the present study (620 min per week) (Jayanthi et al., 2015). However, it was previously reported that early specialization in a single sport increases the risk of sustaining acute and overuse injuries, burnout, and drop out of sports (DiFiori et al., 2014). Of note, the level of sport specialization can be classified as low, moderate, or high depending on the following three factors: (i) participate in year-round training (greater than 8 months per year); (ii) select a main sport, and (iii) quit all other sports to focus on the one main sport only (Myer et al., 2015). According to this classification scheme, all ESC students show a moderate-to-high degree of sports specialization (Jayanthi et al., 2015; Myer et al., 2015). With reference to Jayanthi and colleagues (Jayanthi et al., 2015), ESC's level of sport specialization may increase their risk of sustaining injuries. Of note, the risk of sustaining serious overuse injuries in youth athletes doubles if athletes participate in more hours of sports training per week than number of age in years (OR = 2.07) (Jayanthi et al., 2015). In this study, ESC students mean age was 9.5 years and they exercised on average 10.3 h per week which indicates that injury risk was increased. However, no injuries were reported for the ESC over the course of the 1-year training intervention which is further supported by high adherence rates of 95.9% in sport-specific training and PE. Even though we did not detect any injuries in this study despite the applied high training volumes, coaches and PE teachers are advised to keep training volumes on age and maturation adjusted levels and to conduct neuromuscular training programs to prevent injuries.

### Strengths and limitations

The strengths of this study include the long-term intervention type, the special sample of youth athletes who conducted sport-specialized training at a relatively young age, the detailed training documentation of each conducted training session and PE class over a period of 1 year, and the deduced implications for a structured LTAD. The main limitation of this study was the absence of randomization on a class or school level. However, a randomization process was not feasible because of the unique sports-specific school program. In addition, ESC students started their sport-specific training 6 month prior to our baseline tests. The previous experiences in sport-specific training and the absence of the randomization process resulted in the observed between-group baseline differences.

## Conclusions

In summary, the present study revealed that ESC compared to RC showed better performances in physical fitness, less relative body fat mass and more relative skeletal muscle mass but no significant baseline differences in cognitive and academic performances. The reported sport-specific training in combination with physical education in the ESC resulted in a high mean training volume of 620 min per week and a medium training intensity of 4.8 points on a visual analog scale. We can conclude that ESC's training program is feasible and safe with no training-related injuries and high adherence rates (96%). After the 1-year sport-specific training in combination with physical education, performances in physical fitness were maintained or even increased in the ESC compared to the RC while there were no negative effects of ESC's high training volumes on cognitive and/or academic performances. Thus, it is concluded that sport-specific training in combination with regular school demands promotes physical fitness development and does not impede youth athletes' cognitive and academic performances. The results of the present study represents an example of a sport-specific training program that can be implemented at a relatively young age in schools and sports with early peak performances during the stages of LTAD. Our findings are crucial for parents and policy makers responsible for the well-being of their children. It has to be noted though that more longitudinal studies (>2 years) are needed to achieve in-depths knowledge on the development of youth athletes physical fitness and cognitive/academic performances during the stages of LTAD.

## Ethics statement

This study was carried out in accordance with the recommendations of the ethical committee, University of Potsdam, Germany. All subjects gave written informed consent in accordance with the Declaration of Helsinki. The protocol was approved by the local ethical committee of the University of Potsdam, Germany.

## Author contributions

UG was involved in the design of the study, data collection and analysis, statistical computation, and the writing of the manuscript. RB was involved in the design of the study, implementation of the intervention, data collection and analysis, statistical computation, and the writing of the manuscript.

### Conflict of interest statement

The authors declare that the research was conducted in the absence of any commercial or financial relationships that could be construed as a potential conflict of interest.
